# Anti-glomerular Basement Membrane Disease After Diagnosis of Immunoglobulin A Nephropathy: A Case Report

**DOI:** 10.7759/cureus.39737

**Published:** 2023-05-30

**Authors:** Takahiro Matsuno, Toshiya Okumura

**Affiliations:** 1 Nephrology, Komatsu Sophia Hospital, Komatsu, JPN; 2 Nephrology, Tonami General Hospital, Tonami, JPN

**Keywords:** glomerulonephritis, antibodies, autoimmune disease, immunoglobin a nephropathy, anti-glomerular basement membrane disease

## Abstract

Anti-glomerular basement membrane (anti-GBM) disease has one of the worst prognoses of nephritis and is rarely associated with other forms of glomerulonephritis. In this report, we present the case of a 76-year-old man who developed anti-GBM disease four months after being diagnosed with IgA nephropathy (IgAN). To our knowledge, although there have been several reports of IgAN combined with anti-GBM disease, there have been no cases in which we were able to confirm that the anti-GBM antibody titer changed from negative to positive over the disease course. This case suggests that even patients with previously diagnosed chronic glomerulonephritis, including IgAN, and an unusually rapid clinical course should be evaluated for the presence of autoantibodies to exclude overlapping autoimmune diseases.

## Introduction

Anti-glomerular basement membrane (anti-GBM) disease is a rare autoimmune disease characterized by positive anti-GBM antibodies and linear deposition of IgG along the GBM [1]. Due to its rarity, no definitive statement on the incidence of anti-GBM disease is available. In European populations, it is often said to be less than 1 per million, but this is not certain [2]. Complicated cases of this disease have been reported, including cases that were related to anti-neutrophil cytoplasmic antibody (ANCA) [3,4] and membranous nephropathy [5,6]. However, IgA nephropathy (IgAN) cases are rare [7]. Here, we report a case in which IgAN was diagnosed by renal biopsy; the patient was negative for anti-GBM antibodies at that time; approximately four months later, he was positive for anti-GBM antibodies, and rapidly progressive glomerulonephritis developed.

## Case presentation

**Figure 1:**
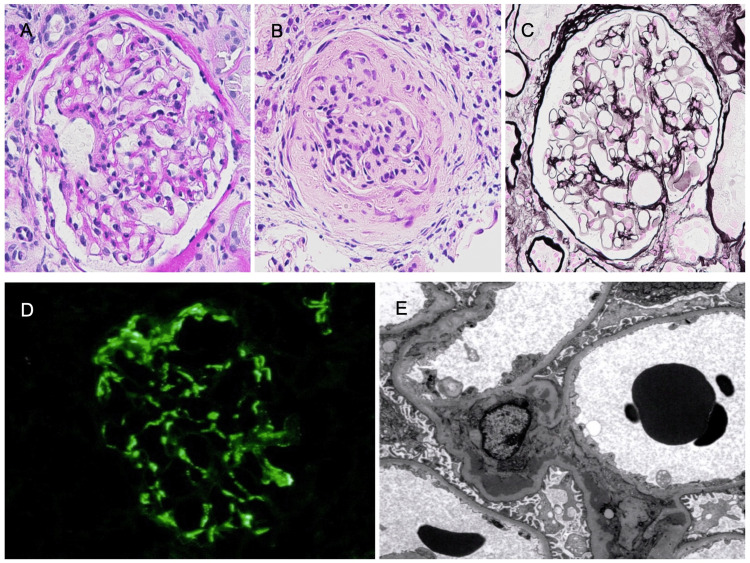
Histopathology of the renal biopsy (A) Mesangial cells and matrix were increased (PAS stain; 400x). (B) One fibrous crescent was observed (PAS stain; 400x). (C) No basement membrane doubling or spike formation was observed (PAM stain; 400x). (D) IgA was strongly deposited in the mesangium (IF). (E) Deposits were seen in the mesangial area. No thickening or thinning of GBM or tear image was observed (EM; 3000x). IgA: immunoglobulin A; PAS: periodic acid-Schiff; PAM: periodic acid-methenamine-silver; IF: immunofluorescence; EM: electron microscope

A 76-year-old Japanese man with hypertension who had been visiting his local doctor was referred to our hospital in June 2019 because of positive urine protein and occult blood. Urinalysis showed urinary protein 2+ and urinary occult blood 3+, and urinary sediment showed erythrocyte levels of 50-99/high power field (HPF). No red blood cell casts or white blood cell casts were observed. Blood tests showed a creatinine (Cr) level of 0.89 mg/dL (reference range: 0.65 to 1.09 mg/dL) and IgA levels of 450 mg/dL (reference range: 90 to 400 mg/dL). The patient was negative for Complement C3, anti-nuclear antibody (ANA), and ANCA. A renal biopsy was performed in July 2019. Light microscopy revealed 18 glomeruli, including two all-nodular sclerosing glomeruli. Five glomeruli with increased mesangial cells and substrates, as well as one fibrous crescent, were observed (Figure 1). Using the Oxford Classification of IgAN, the patient’s biopsy showed mesangial hypercellularity (M0: ≤50% of the glomeruli showing mesangial hypercellularity) and endocapillary hypercellularity (E1) without segmental sclerosis (S0), interstitial fibrosis/ tubular atrophy (T0), or crescents (C0). Immunofluorescence staining showed mesangial deposits of IgA (IgA: [++] mes), whereas electron microscopy showed deposits in the mesangial area, no thickening or thinning of the GBM, and no fracture (Figure 1). Accordingly, IgAN was diagnosed. The treatment plan was to continue with angiotensin II receptor blockers (ARB), which had been previously prescribed for hypertension.

Thereafter, the patient was followed up during outpatient visits. In November 2019, a fever of 37.5°C and general malaise were observed, and the patient visited our clinic without an appointment. Physical examination revealed lower leg edema. Urinalysis showed that his condition was worsening, and blood samples showed an elevated inflammatory response and a Cr of 5.18 mg/dL (Table 1). He was urgently admitted to our department for close examination and treatment of progressive renal dysfunction and elevated inflammatory response.

**Table 1:** Laboratory findings on admission MPO-ANCA: myeloperoxidase-anti-neutrophil cytoplasmic antibodies; PR3-ANCA: proteinase-3-anti-neutrophil cytoplasmic antibodies; Cr: creatinine; Na: sodium; K: potassium; Cl: chloride; CRP: C-reactive protein; IgG: immunoglobulin G; IgA: immunoglobulin A; IgM: immunoglobulin M; ANA: anti-nuclear antibody; RBC: red blood cells; WBC: white blood cells

Laboratory test	Unit	Patient’s laboratory values	Reference ranges
White blood cell	/μL	10000	3300-8600
Hemoglobin	g/dL	10.1	13.7-16.8
Plates	/μL	34.9×10^4^	15.8×10^4^-34.8×10^4^
Total protein	g/dL	6.0	6.9-8.4
Albumin	g/dL	2.4	3.9-5.2
Urea nitrogen	mg/dL	57.5	8-21
Cr	mg/dL	5.18	0.65-1.09
Na	mEq/L	140	135-146
K	mEq/L	4.1	3.7-4.8
Cl	mEq/L	103	101-109
CRP	mg/dL	13.7	<0.3
IgG	mg/dL	1067	870-1700
IgA	mg/dL	468	90-400
IgM	mg/dL	76	33-190
C3	mg/dL	164	73-138
C4	mg/dL	33	11-31
CH50	U/mL	50	30-45
ANA	SP	40	<40
MPO-ANCA	EU	<10	<10
PR3-ANCA	EU	<10	<10
Anti-GBM antibody titer	U/mL	485.7	<3.0
Urinalysis			
Protein		3+	Negative
Blood		3+	Negative
RBC	/HF	>200	Negative
WBC	/HF	5-9	Negative

**Figure 2:**
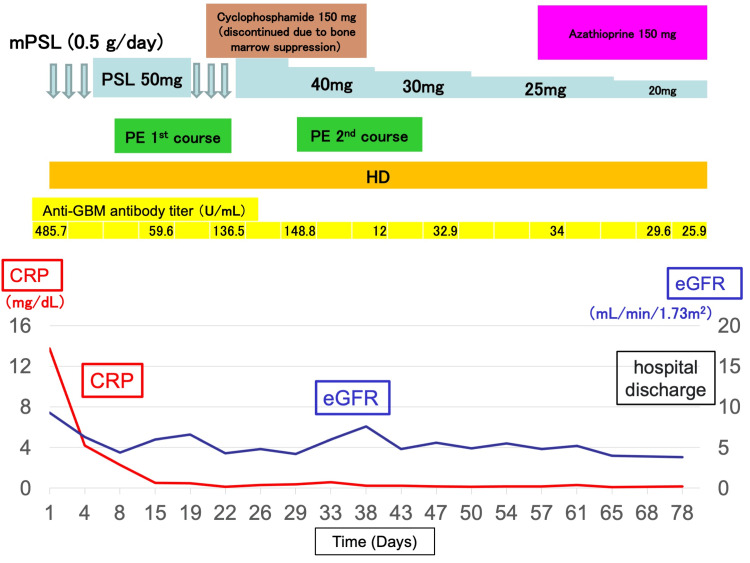
Clinical course mPSL: methylprednisolone; PE: plasma exchange; HD: hemodialysis; GBM: glomerular basement membrane; CRP: C-reactive protein; eGFR: estimated glomerular filtration rate

Worsening renal function was suspected to be an aggravation of the nephritis, and steroid pulse therapy was initiated as soon as the patient was admitted. Simultaneously, a urinary tract infection could not be completely excluded; therefore, the patient was treated with ceftriaxone 2 g/day. Prednisolone (PSL) 50 mg was started on the fifth day as post-treatment. On the eighth day, the anti-GBM antibody was found to be positive at 485.7 U/mL, and a diagnosis of anti-GBM disease was made. Plasma exchange (PE) therapy was initiated and performed seven times during a 14-day course. Hemodialysis was performed simultaneously. The anti-GBM antibody titer improved to 59.6 U/mL, but the patient remained positive for anti-GBM, and a second dose of steroid pulse therapy was administered on the eighth day. The patient was treated with cyclophosphamide; 150 mg was administered from the 19^th^ day. His anti-GBM antibody titer increased again (136.5 U/mL), and a second course of PE was administered from the 30^th^ day of the disease. On the 37^th^ day, a blood sample was collected; pancytopenia progressed, and the drug was discontinued based on the suspicion of myelosuppression caused by cyclophosphamide. After the second course of PE, the anti-GBM antibody titer showed a gradual downward trend; therefore, PSL was tapered. From the 60^th^ day, azathioprine 150 mg was added, and PSL was reduced to 20 mg. Thereafter, there were no apparent relapse findings, but renal function did not improve, and he required permanent hemodialysis. The patient was discharged home on the 78^th^ day and remained on maintenance dialysis at our hospital after discharge (Figure 2).

## Discussion

Here, we report a case of anti-GBM disease complicated by IgAN approximately four months after diagnosis. Although several cases of anti-GBM nephritis associated with IgAN have been reported, no case has been reported in which the GBM antibody titer was negative at the time of IgAN diagnosis.

The mechanism by which IgAN is complicated by anti-GBM diseases remains unclear. Various hypotheses have been proposed to explain the phenomenon. IgA-associated immune complexes promote immunological and inflammatory events that lead to GBM antigen exposure and anti-GBM antibody production [8]. Secondly, it has been hypothesized that abnormal IgA deposition along the GBM triggers the formation of new antigens, leading to the production of anti-GBM antibodies [9]. Other hypotheses propose that anti-GBM antibodies alter the permeability of GBM, allowing the deposition of circulating immune complexes on the mesangium [9]. However, it is difficult to prove whether the anti-GBM disease in these patients is an incidental complication or secondary to IgAN since no biomarkers have been identified to distinguish primary from secondary anti-GBM disease [10]. However, in the present case, IgAN was confirmed to be negative for anti-GBM antibodies at the time of diagnosis, and renal biopsy showed the aforementioned results, suggesting that IgAN occurred first and that anti-GBM nephritis occurred later. Thus, the final hypothesis may be negative. However, more cases need to be studied.

It has been reported that the overall dialysis dependence rate of anti-GBM disease is 69% and that renal prognosis is relatively good (31%) only in patients with IgAN complications [11]. Unfortunately, the patient died due to renal failure and required maintenance dialysis. The reason for this was the high titer of the anti-GBM antibody. High titers of anti-GBM antibodies are reportedly associated with severe disease and poor renal prognosis [12]. The highest antibody titer reported thus far among patients with IgAN-associated anti-GBM disease is 258.3 U/mL, and the titer in the present case was almost twice that level.

The rarity of this case is that the patient developed anti-GBM nephritis approximately four months after the diagnosis of IgAN. Furthermore, the anti-GBM antibody titer increased from negative to 485.7 U/mL during the four-month period, which was unprecedented. Even if IgAN has been diagnosed in the past, it should not be regarded simply as an acute exacerbation; other factors should also be thoroughly investigated.

## Conclusions

Herein, we describe a case of IgAN followed by anti-glioblastoma nephritis. When renal function worsened, we first considered IgAN exacerbation. However, this was not the case. Even if the patient has already been diagnosed with chronic glomerulonephritis, including IgAN, autoantibodies should be confirmed again according to the clinical course.
